# Social Inequities in Access to Dental Care in Australian Adults over Time

**DOI:** 10.1177/23800844241253274

**Published:** 2024-06-13

**Authors:** L.M. Jamieson, L. Luzzi, G.C. Mejia, S. Chrisopoulos, X. Ju

**Affiliations:** Australian Research Centre for Population Oral Health, Adelaide Dental School, the University of Adelaide, Adelaide, South Australia, Australia

**Keywords:** social inequality, equivalized household income, dental caries experience, untreated dental caries

## Abstract

**Introduction:**

Social inequities in dental caries are reflected by both inequities in the social structures that contribute to disease severity and inequities in the provision of dental care. This study aimed to describe social differentials in the dental caries experience among Australian adults across a 13-y period and to examine if the highest magnitude of oral health inequities persisted across dental caries prevalence (decayed teeth [DT]) or its management (missing teeth [MT], filled teeth [FT]).

**Methods:**

Data were from 2 population-based cross-sectional surveys of Australian adult oral health conducted in 2004–2006 (National Survey of Adult Oral Health–1 [NSAOH-1], n = 5,505) and 2017–2018 (NSAOH-2, n = 5,022). In each survey, representative samples of adults were obtained through a 3-stage, stratified sample design within metropolitan and regional areas in each state/territory. Clinical outcomes included the prevalence and mean of DT, MT, FT, and DMFT. Equivalized household income was grouped into approximately quartiles from low to high.

**Results:**

Across all income quartiles, the mean DT and % DT >0 was higher in NSAOH-2 than in NSAOH-1. The increase in prevalence was highest in the third highest income group (prevalence difference [PD] = 8.4, from 24.1 to 32.5). Similarly, % MT >0 was lower in NSAOH-2 than in NSAOH-1 across all income groups, with the decrease most marked for the lowest income group (PD = −6.5, from 74.1 to 67.8). Across all income quartiles, % FT >0 was lower in NSAOH-2 than in NSAOH-1. The decrease was the most marked for the lowest income group (PD = −8.9, from 81.1 to 72.2).

**Conclusion:**

The findings confirm that although oral health inequities decreased for the most extreme management outcome of dental caries (MT), inequities increased for experience of that disease (DT) and the more conservative management of dental caries (FT). For all D, M, and F components (DMFT), inequities between the lowest and highest household income groups increased from 2004–2006 to 2017–2018.

**Knowledge Transfer Statement:**

This study found that social inequities in oral health (experience of untreated dental caries and missing teeth) increased between the most socially advantaged and disadvantaged groups between 2004–2006 and 2017–2018. This suggests that models of dental service provision in Australia are increasingly benefitting those who can afford and access the care and who arguably need the services less than their less socially advantaged counterparts do.

## Introduction

Social inequities in oral health are reflected by both inequities in the social structures that contribute to disease severity and inequities in the provision of dental care (extraction vs. restoration; Watt et al. 2019). The two often occur simultaneously, with the greatest magnitude of dental disease severity occurring among the socially disadvantaged. Such groups, many of whom live with overlaying patterns of intersectionality, may present late to a given dental provider for a multitude of reasons, and for a multitude of consequential reasons (bias in dental service providers, financial constraints), they may not receive care that is conducive to long-term maintenance of oral structures (including the periodontium; Ghanbarzadegan et al. 2023). In Australia, research demonstrates that oral health inequities are more apparent in the provision of care to treat dental diseases than in measures of oral diseases itself (Mejia et al. 2014).

Measures of oral disease provide a lens into the accumulative nature of dental disease across the life course. At any given time, a dental assessment may detect dental caries due to dental checkup, service provision that does not lead to optimal maintenance of the dental arch (teeth that have been extracted), or optimal dental care (teeth that have been restored, either with a restoration or more complex root canal treatment, crown, bridge, or implant; Ruiz et al. 2023).

The predominant model of dental service provision for adults in Australia is through the private sector (Council of Australian Governments Health Council [COAG] 2015), with about half (51%) of the adult Australian population having dental insurance and only 30% of participants in the 2017–2018 National Study of Adult Oral Health being eligible for means-tested dental service provision through the public health sector (Australian Research Centre for Population Oral Health [ARCPOH] 2019). Dental service provision through the public sector has a number of limitations, including wait lists (up to 2 y in some jurisdictions), limited dental care treatment options and locations, and co-payments (Australian Government Productivity Commission 2019). These rationings act to both suppress demand and encourage treatment rather than prevention dental attendance, which frequently translates into poor oral health outcomes.

To address disparities in oral health, an understanding of whether such inequities are due to experience of oral disease per se or treatment of that disease, oral epidemiological assessments at a population level over time are necessary. This is important to inform and facilitate priorities addressed in Australia’s National Oral Health Plan 2015–2024 (COAG 2015) to determine whether oral health inequities are greater in dental disease experience (prevalence of disease) or in the treatment of that disease (inequities in dental service provision). The aim of this study was to describe social differentials in dental caries experience among Australian adults across a 13-y period and to examine whether the highest magnitude of oral health inequities persisted across dental caries (decayed teeth) or its management (missing teeth and filled teeth).

## Methods

This study is reported according to STROBE (Strengthening the Reporting of Observational Studies in Epidemiology) guidelines.

### Study Design and Sample Selection

Data were from 2 population-based cross-sectional surveys of Australian adult oral health conducted in 2004–2006 (National Survey of Adult Oral Health–1; NSAOH-1) and 2017–2018 (NSAOH-2; Chrisopoulos et al. 2020; Slade et al. 2007). In each survey, representative samples of adults were drawn through a 3-stage, stratified sample design within metropolitan and regional areas in each Australian state/territory. The first stage selected a sample of postcodes from all in-scope postcodes. The second stage selected households within sampled postcodes, with adults aged 15 y and older being randomly selected from each sample household to participate in the final stage. Data were weighted following standard procedures for clustered samples. Both NSAOH-1 and NSAOH-2 were reviewed and approved by the University of Adelaide Human Research Ethics Committee.

Self-reported information about oral health and related characteristics were collected using a computer-assisted telephone interview (CATI) in 2004–2006 and CATI or online questionnaire in 2017–2018. Dentate participants were invited to receive a standardized oral epidemiological examination. Information about dental clinical status was collected during examinations, which were conducted by calibrated dental providers. All examiners were tested in the field against a gold standard examiner to estimate interexaminer reliability. The intraclass correlation coefficient for the number of teeth present was 1.00; the number of decayed (DT; referred to International Caries Detection and Assessment System score >2), missing (MT), and filled (FT) teeth (DMFT) was 0.96.

### Outcome Variables

Dental caries (prevalence and mean) was the outcome variable, assessed using the proportion of people with decayed teeth (% DT >0), missing teeth (% MT >0), filled teeth (% FT >0), and overall decayed, missing, and filled teeth (% DMFT >0). In addition, mean DT, MT, FT, and DMFT were calculated.

### Explanatory Variables

Age was grouped into 15 to 34 y, 35 to 54 y, and 55+ y. Sex was classified as male or female. Residential location was categorized into major city or regional/remote. Culturally and linguistically diverse (CALD) status was identified based on English being not the primary language spoken at home and country of birth not being Australia. Participants were thus categorized as being either (1) born overseas and not speaking English as the primary language at home or (2) all others (born overseas and English primary language at home, born in Australia and English not primary language at home, born in Australia and English primary language at home). Household income was divided by an equivalence factor using the Organisation for Economic Cooperation and Development modified scale (Hagenaars et al. 1994). The equivalence factor is the sum of allocated points to household members (i.e., 1 point for the first adult, 0.5 points to each additional person aged 15 y and older, and 0.3 to each child younger than 15 y). Equivalized household income was then grouped into approximate quartiles from low to high. Last dental visit was derived from the question “How long ago did you last see a dental professional about your teeth, dentures, or gums?” with responses dichotomized into “less than 1 y” and “1 y or more.” Dental insurance was derived from 3 questions: (1) “Do you have a private health insurance other than Medicare?” If the response was “yes” or “don’t know,” participants were asked, “What type of private medical insurance do you have?” If people reported having extra or responded “don’t know,” they were asked, “Does your private health insurance provide cover dental services?” Those who responded “yes” were classified as having private dental insurance.

### Data Analysis

Data files were managed and summary variables computed using SAS software version 9.4 (SAS 9.4, SAS Institute Inc.). Forest plots were drawn using Stata 14.1 software (StataCorp). Weights were used to account for the complex sampling methodology of the surveys.

The analysis began with the computation of univariate statistics describing prevalence (% and 95% confidence interval [CI] of DT >0, MT >0, FT >0, DMFT >0) and average (mean and 95% CI of D, M, F, and DMFT) of dental caries experience across 2 time points, stratified by covariates of interest. Statistically significant differences were denoted by 95% CIs that did not overlap. Absolute prevalence differences (PDs), mean differences (MDs), and their corresponding 95% CIs were estimated to examine household income inequality by using random effects meta-analysis, which was adjusted for age and sex to the average covariate distribution of the 4 household income groups combined. The I^2^ statistic—the percentage of total variation across income groups attributed to heterogeneity rather than chance—was used to quantify the heterogeneity among groups (Borenstein et al. 2009, 2017). An I^2^ value >75% and a *P* value <0.05 were considered indicative of significant (high) heterogeneity.

## Results

In NSAOH-1, data were available for 5,505 participants. In NSAOH-2, data were available for 5,022 participants. Across the 2 time points, statistically significant differences were observed for those identifying as CALD (9.7% vs. 14.4%), lowest household income quartile (20.3% vs. 30.4%), last dental visit 12+ mo ago (40.6% vs. 43.6%), and dental insurance status (45.6% vs. 51.1%; Table 1). In NSAOH-1, the proportion with 1 or more teeth that was decayed, missing, or filled was 26%, 61%, and 83% respectively. The total % DMFT >0 was 90%. In NSAOH-2, the proportion with 1 or more teeth that was decayed, missing, or filled was 31%, 61%, and 77%, respectively. The total % DMFT >0 was 89%. Across the 2 time points, statistically significant differences were observed for % DT >0 (increased) and % FT >0 (decreased). When stratified by covariates, there were marked increased inequities observed for the CALD group with % DT >0 between the 2 time points (3% difference in NSAOH-1 compared with 14% difference in NSAOH-2). Similar inequities were observed among those without dental insurance; from NSAOH-1 to NSAOH-2, % DT >0 increased from 31% to 39% and % FT >0 decreased from 82% to 74%.

**Table 1.** Changes in % DT **>**0, % MT **>**0, and % FT **>**0 Teeth among Australian Adults across Time, NSAOH 2004–2006 and NSAOH 2017–2018, Weighted Estimates.

		% DT >0	% MT >0	% FT >0	% DMFT >0
	% (95% CI)	Mean (95% CI)			
	NSAOH 2004–2006 (Total *N* = 14,123, exam *n* = 5,505)				
Total	100	25.5 (23.6–27.3)	61.0 (58.5–63.5)	83.4 (81.5–85.3)	90.1 (88.5–91.7)
Age group (y)					
15 to 34	34.8 (33.5–36.1)	25.8 (22.2–29.4)	25.8 (21.7–30.0)	64.8 (60.6–69.0)	75.9 (72.2–79.6)
35 to 54	35.5 (34.4–36.6)	27.1 (24.5–29.7)	70.7 (67.8–73.6)	94.6 (93.2–96.0)	97.6 (96.6–98.6)
55+	29.7 (28.5–30.9)	22.5 (20.2–24.8)	98.1 (97.1–99.1)	94.2 (92.8–95.7)	99.8 (99.5–100.0)
Sex					
Male	49.4 (48.3–50.5)	28.2 (25.4–31.0)	61.8 (58.1–65.6)	81.8 (78.6–84.9)	89.2 (86.7–91.8)
Female	50.6 (49.5–51.7)	22.7 (20.4–24.9)	60.2 (57.3–63.0)	85.1 (83.0–87.1)	90.9 (89.2–92.6)
Residential location					
Regional/remote	33.1 (29.8–36.3)	30.3 (27.2–33.4)	67.8 (63.7–72.0)	84.7 (81.3–88.0)	91.1 (88.4–93.7)
Major city	66.9 (63.7–70.2)	23.1 (20.8–25.4)	57.7 (54.7–60.8)	82.8 (80.4–85.2)	89.6 (87.6–91.6)
CALD status^a^					
CALD	9.7 (8.7–10.8)	28.7 (22.4–35.0)	62.2 (54.4–69.9)	82.9 (76.8–89.0)	91.7 (86.5–96.8)
All others	90.3 (89.2–91.3)	25.1 (23.2–27.1)	60.9 (58.3–63.4)	83.5 (81.4–85.5)	89.9 (88.2–91.6)
Equivalized household income					
Quartile 1 (low)	20.3 (19.2–21.4)	35.0 (30.5–39.5)	76.6 (71.9–81.2)	81.9 (77.6–86.1)	93.6 (90.3–97.0)
Quartile 2	28.2 (27.1–29.2)	26.0 (22.7–29.4)	68.6 (64.4–72.8)	86.0 (82.5–89.6)	91.4 (87.5–93.3)
Quartile 3	29.2 (28.0–30.4)	23.9 (20.2–27.7)	58.1 (53.5–62.8)	83.2 (79.0–87.4)	90.4 (87.5–93.3)
Quartile 4 (high)	22.3 (21.1–23.5)	19.8 (16.5–23.2)	55.2 (50.9–59.4)	90.0 (87.3–92.7)	92.9 (90.7–95.1)
Last dental visit					
12+ mo ago	40.6 (39.5–41.8)	32.0 (29.0–35.0)	52.0 (48.5–55.5)	75.5 (72.2–78.8)	86.9 (84.1–89.7)
<12 mo ago	59.4 (58.2–60.5)	20.8 (18.5–23.1)	67.4 (64.5–70.3)	89.0 (86.9–91.1)	92.3 (90.5–94.1)
Dental insurance					
No	54.4 (52.9–56.0)	31.1 (28.5–33.8)	62.2 (59.0–65.4)	81.6 (78.8–84.3)	90.8 (88.7–92.9)
Yes	45.6 (44.0–47.1)	19.4 (16.8–22.0)	61.7 (58.1–65.2)	87.5 (85.1–89.9)	91.3 (89.1–93.4)
	NSAOH 2017–2018 (Total *N* = 15,731, exam *n* = 5,022)				
Total	100	31.1 (29.5–34.7)	60.6 (57.8–63.3)	76.9 (74.6–79.2)	89.3 (87.6–90.9)
Age group (y)					
15 to 34	34.5 (33.4–35.7)	30.3 (25.4–35.1)	19.9 (15.9–23.8)	61.0 (56.3–65.7)	74.4 (70.3–78.6)
35 to 54	32.6 (31.5–33.6)	35.4 (31.2–39.7)	70.4 (66.4–74.5)	85.1 (81.8–88.4)	95.4 (93.7–97.1)
55+	32.9 (32.0–33.8)	30.5 (26.8–34.3)	97.9 (96.2–99.5)	86.7 (84.0–89.5)	100.0 (100.0–100.0)
	NSAOH 2017–2018 (Total *N* = 15,731, exam *n* = 5,022)				
Sex					
Male	49.2 (48.1–50.4)	34.7 (30.0–38.4)	59.0 (55.1–62.9)	73.9 (70.3–77.5)	87.6 (84.9–90.2)
Female	50.8 (49.6–51.9)	29.5 (26.1–32.9)	62.2 (58.6–65.7)	79.9 (77.1–82.7)	90.9 (88.9–92.9)
Residential location					
Regional/remote	28.2 (25.1–31.3)	33.1 (29.1–37.1)	67.4 (62.7–72.1)	78.2 (75.3–81.1)	91.8 (89.4–94.3)
Major city	71.8 (68.7–74.9)	31.7 (28.4–35.0)	58.0 (54.7–61.3)	76.4 (73.4–79.4)	88.3 (86.2–90.4)
CALD status^a^					
CALD	14.4 (13.0–15.9)	44.0 (36.3–51.7)	62.3 (55.3–69.4)	74.4 (66.6–82.3)	92.0 (88.0–96.0)
All others	85.6 (84.1–87.0)	30.0 (27.4–32.7)	60.3 (57.3–63.3)	77.3 (75.0–79.7)	88.8 (87.0–90.6)
Equivalized household income					
Quartile 1 (low)	30.4 (28.9–31.8)	38.5 (33.3–43.6)	70.9 (65.5–76.2)	73.2 (68.4–78.0)	90.1 (86.7–93.5)
Quartile 2	23.8 (22.7–24.8)	32.2 (27.0–37.3)	66.9 (61.8–72.0)	77.8 (73.3–82.3)	90.8 (87.5–94.2)
Quartile 3	25.2 (24.1–26.3)	32.4 (28.0–36.9)	59.4 (54.6–64.3)	82.8 (79.5–86.1)	91.0 (88.5–93.5)
Quartile 4 (high)	20.7 (19.5–21.9)	21.0 (16.5–25.5)	51.3 (45.6–56.9)	84.2 (80.2–88.3)	90.9 (88.1–93.8)
Last dental visit					
12+ mo ago	43.6 (42.4–44.9)	39.2 (35.4–43.1)	57.3 (53.3–61.4)	67.1 (63.2–71.0)	86.5 (83.7–89.3)
<12 mo ago	56.4 (55.1–57.6)	25.3 (22.2–28.5)	63.5 (60.0–67.0)	86.0 (83.7–88.3)	91.7 (89.7–93.8)
Dental insurance					
No	48.9 (47.2–50.5)	38.6 (35.0–42.2)	62.5 (58.8–66.1)	73.9 (70.7–77.1)	89.7 (87.3–92.0)
Yes	51.1 (49.5–52.8)	24.4 (21.2–27.6)	60.0 (56.3–63.6)	81.7 (78.7–84.7)	89.8 (87.6–91.9)

CALD, culturally and linguistically diverse; DT, decayed teeth; FT, filled teeth; MT, missing teeth; NSAOH, National Survey of Adult Oral Health.

a CALD was defined by country of birth not being Australia AND English not primary language spoken at home.

The mean number of D, M, F, and DMFT is presented in Table 2. Overall, the mean number of MT (4.55 vs. 4.41), FT (7.69 vs. 5.95), and DMFT (12.85 vs. 11.21) decreased, but DT (0.61 vs. 0.85) increased from NSAOH-1 to NSAOH-2. The highest mean number of DT in both time points was found in participants in the youngest age group, males, those residing in regional areas, those in the lowest income group, those whose last dental visit was more than 12 mo ago, and those without dental insurance. The highest mean number of MT across both time points occurred in participants in the oldest age group, those residing in regional areas, those in the lowest income group, those whose last dental visit was less than 12 mo ago, and participants without dental insurance, and the lowest number of MT was observed in CALD in NSAOH-2. The lowest mean number of FT in both time points occurred in those in the youngest age group, males, participants in the CALD group, those whose last dental visit was more than 12 mo ago, and those who did not have dental insurance, and the highest mean number of FT was seen in the second lowest income group (Q2) in NSAOH-1. The highest mean number of DMFT in both time points was observed in participants in the oldest age group, females, those residing in regional areas, those in the lowest income group (Q1), those whose last dental visit was less than 12 mo ago, and those with dental insurance in NSAOH-1.

**Table 2.** Changes in D, M, and F Teeth among Australian Adults across Time, NSAOH 2004–2006 and NSAOH 2017–2018, Weighted Estimates.

	DT	MT	FT	DMFT
	Mean (95% CI)			
	NSAOH 2004–2006 (Total *N* = 14,123, exam *n* = 5,505)			
Total	0.61 (0.57–0.65)	4.55 (4.39–4.72)	7.69 (7.52–7.86)	12.85 (12.61–13.10)
Age group (y)				
15 to 34	0.69 (0.59–0.79)	0.79 (0.69–0.88)	3.10 (2.88–3.32)	4.58 (4.29–4.86)
35 to 54	0.64 (0.58–0.71)	3.91 (3.73–4.10)	9.82 (9.58–10.01)	14.37 (14.07–14.68)
55+	0.43 (0.38–0.48)	10.98 (10.68–11.28)	11.28 (11.01–11.6)	22.69 (22.48–22.9)
Sex				
Male	0.70 (0.64–0.77)	4.47 (4.22–4.72)	7.24 (6.98–7.51)	12.42 (12.04–12.80)
Female	0.51 (0.46–0.56)	4.64 (4.43–4.85)	8.14 (7.92–8.37)	13.29 (12.97–13.62)
Residential location				
Regional/remote	0.76 (0.69–0.83)	5.41 (5.14–5.67)	7.87 (7.62–8.13)	14.04 (13.68–14.41)
Major city	0.53 (0.48–0.58)	4.15 (3.94–4.35)	7.61 (7.38–7.83)	12.28 (11.96–12.61)
CALD status^a^				
CALD	0.61 (0.56–0.65)	4.44 (3.86–5.02)	6.66 (6.09–7.23)	11.71 (10.85–12.57)
All others	0.61 (0.56–0.65)	4.57 (4.40–4.73)	7.79 (7.62–7.97)	12.97 (12.71–13.22)
Equivalized household income				
Quartile 1 (low)	0.85 (0.74–0.96)	7.53 (7.05–8.01)	7.24 (6.87–7.62)	15.62 (15.06–16.19)
Quartile 2	0.66 (0.58–0.75)	5.84 (5.49–6.19)	8.27 (7.94–8.61)	14.78 (14.28–15.27)
Quartile 3	0.55 (0.47–0.62)	3.22 (2.99–3.46)	8.17 (7.81–8.53)	11.94 (11.47–12.41)
Quartile 4 (high)	0.43 (0.36–0.49)	3.20 (2.95–3.44)	8.17 (7.84–8.50)	11.79 (11.34–12.25)
Last dental visit				
12+ mo ago	0.80 (0.73–0.88)	4.07 (3.79–4.34)	5.73 (5.49–5.97)	10.61 (10.23–10.98)
<12 mo ago	0.47 (0.42–0.51)	4.90 (4.70–5.10)	9.08 (8.86–9.31)	14.45 (14.13–14.76)
Dental insurance				
No	0.81 (0.74–0.88)	5.04 (4.79–5.29)	6.79 (6.57–7.01)	12.64 (12.30–12.99)
Yes	0.38 (0.34–0.42)	4.17 (3.96–4.38)	8.99 (8.74–9.25)	13.55 (13.19–13.90)
	NSAOH 2017–2018 (Total *N* = 15,731, exam *n* = 5,022)			
Total	0.85 (0.80–0.90)	4.41 (4.25–4.57)	5.95 (5.79–6.11)	11.21 (10.96–11.45)
Age group (y)				
15 to 34	0.91 (0.79–1.04)	0.60 (0.51–0.69)	2.62 (2.44–2.81)	4.13 (3.88–4.38)
35 to 54	0.88 (0.80–0.96)	3.63 (3.44–3.82)	5.81 (5.58–6.05)	10.32 (9.99–10.66)
55+	0.74 (0.66–0.82)	9.78 (9.49–10.07)	10.02 (9.76–10.3)	20.54 (20.27–20.8)
	NSAOH 2017–2018 (Total *N* = 15,731, exam *n* = 5,022)			
Sex				
Male	0.94 (0.86–1.02)	4.22 (3.98–4.46)	5.20 (4.99–5.42)	10.37 (10.01–10.73)
Female	0.76 (0.69–0.83)	4.59 (4.37–4.81)	6.68 (6.45–6.90)	12.03 (11.69–12.37)
Residential location				
Regional/remote	0.89 (0.80–0.98)	5.39 (5.11–5.66)	6.21 (5.96–6.45)	12.49 (12.10–12.88)
Major city	0.83 (0.76–0.90)	4.04 (3.84–4.25)	5.85 (5.64–6.06)	10.73 (10.41–11.04)
CALD status^a^				
CALD	0.87 (0.75–0.99)	3.60 (3.16–4.04)	5.04 (4.62–5.46)	9.51 (8.87–10.15)
All others	0.85 (0.79–0.90)	4.55 (4.38–4.72)	6.10 (5.93–6.27)	11.50 (11.23–11.77)
Equivalized household income				
Quartile 1 (low)	1.04 (0.91–1.17)	5.86 (5.46–6.25)	6.08 (5.72–6.43)	12.98 (12.42–13.53)
Quartile 2	0.99 (0.86–1.12)	5.37 (4.99–5.76)	6.50 (6.12–6.87)	12.86 (12.28–13.44)
Quartile 3	0.75 (0.65–0.86)	3.47 (3.19–3.76)	6.36 (6.03–6.70)	10.60 (10.10–11.08)
Quartile 4 (high)	0.36 (0.30–0.41)	2.92 (2.65–3.19)	6.10 (5.78–6.42)	9.38 (8.90–9.86)
Last dental visit				
12+ mo ago	1.11 (1.01–1.20)	3.98 (3.75–4.22)	4.26 (4.06–4.46)	9.35 (9.01–9.69)
<12 mo ago	0.60 (0.55–0.66)	4.81 (4.58–5.03)	7.51 (7.28–7.73)	12.92 (12.57–13.26)
Dental insurance				
No	1.09 (1.00–1.17)	5.02 (4.75–5.27)	5.21 (5.00–5.43)	11.31 (10.95–11.68)
Yes	0.53 (0.47–0.58)	3.86 (3.66–4.05)	7.02 (6.78–7.25)	11.40 (11.06–11.74)

CALD, culturally and linguistically diverse; DT, decayed teeth; MT, missing teeth; FT, filled teeth; NSAOH, National Survey of Adult Oral Health.

a CALD defined by country of birth not being Australia AND English not primary language spoken at home.

After adjusting for age and sex, across all income quartiles, the mean number of FT and DMFT decreased in all income groups from NSAOH-1 to NSAOH-2 (Table 3). For FT, the greatest decrease was in the highest income group (Q4; MD = 2.0, from 1.6 to 2.5), and the lowest decrease was in the lowest income group (Q1; MD = 1.3, MD = 0.8 to 1.9). For DMFT, the greatest decrease was in the lowest income group (Q1; MD = 2.7, from 1.9 to 3.5), and the lowest decrease was in the third highest income group (Q3; MD = 1.5, MD = 0.8 to 2.1). The mean number of MT decreased in the lowest income group (Q1) from NSAOH-1 to NSAOH-2 (MD = 1.5, from 0.9 to 2.1). The mean number of DT increased in the second- and third-income groups (Q2 and Q3) from NSAOH-1 to NSAOH-2, MD = −0.3 (95% CI: −0.5 to −0.2) and MD = −0.2 (95% CI: −0.3 to −0.1), respectively.

**Table 3.** Adjusted Mean and Absolute Mean Difference for Clinical Oral Health Characteristics by Income Status.

	DT		MT	
	Mean (95% CI)	MD (95% CI)	Mean (95% CI)	MD (95% CI)
Household income Q1 (low)				
NSAOH-1	0.9 (0.8 to 1.0)	−0.2 (−0.4 to 0.0)	**6.9 (6.5 to 7.4)**	1.5 (0.9 to 2.1)
NSAOH-2	1.0 (0.9 to 1.2)	Ref	**5.4 (5.0 to 5.8)**	Ref
Household income Q2				
NSAOH-1	**0.7 (0.6 to 0.8)**	−0.3 (−0.5 to −0.2)	5.4 (5.0 to 5.7)	0.5 (−0.0 to 1.0)
NSAOH-2	**1.0 (0.9 to 1.2)**	Ref	4.9 (4.5 to 5.3)	Ref
Household income Q3				
NSAOH-1	**0.6 (0.5 to 0.6)**	−0.2 (−0.3 to −0.1)	3.0 (2.8 to 3.3)	−0.2 (−0.5 to 0.2)
NSAOH-2	**0.8 (0.7 to 0.9)**	Ref	3.2 (2.9 to 3.5)	Ref
Household income Q4 (high)				
NSAOH-1	0.4 (0.4 to 0.5)	0.1 (−0.0 to 0.2)	3.0 (2.7 to 3.2)	0.3 (−0.1 to 0.6)
NSAOH-2	0.4 (0.3 to 0.4)	Ref	2.7 (2.5 to 3.0)	Ref
	FT		DMFT	
	Mean (95% CI)	MD (95% CI)	Mean (95% CI)	MD (95% CI)
Household income Q1 (low)				
NSAOH-1	**7.1 (6.7 to 7.5)**	1.3 (0.8 to 1.9)	**14.9 (14.3 to 15.5)**	2.7 (1.9 to 3.5)
NSAOH-2	**5.8 (5.4 to 6.1)**	Ref	**12.2 (11.6 to 12.7)**	Ref
Household income Q2				
NSAOH-1	**8.0 (7.6 to 8.3)**	1.9 (1.4 to 2.4)	**14.0 (13.5 to 14.5)**	2.0 (1.2 to 2.7)
NSAOH-2	**6.1 (5.8 to 6.5)**	Ref	**12.1 (11.5 to 12.6)**	Ref
Household income Q3				
NSAOH-1	**7.9 (7.6 to 8.3)**	1.9 (1.4 to 2.3)	**11.5 (11.1 to 12.0)**	1.5 (0.8 to 2.1)
NSAOH-2	**6.1 (5.8 to 6.4)**	Ref	**10.1 (9.6 to 10.5)**	Ref
Household income Q4 (high)				
NSAOH-1	**7.9 (7.6 to 8.2)**	2.0 (1.6 to 2.5)	**11.3 (10.9 to 11.8)**	2.4 (1.7 to 3.0)
NSAOH-2	**5.9 (5.6 to 6.2)**	Ref	**8.9 (8.5 to 9.4)**	Ref

All estimates adjusted for age and sex. 95% CI: confidence interval in parentheses. Estimates in bold denote statistically significant differences. DT, decayed teeth; FT, filled teeth; MD, mean difference; MT, missing teeth; NSAOH, National Survey of Adult Oral Health; Ref, reference.

**Figure 1.**
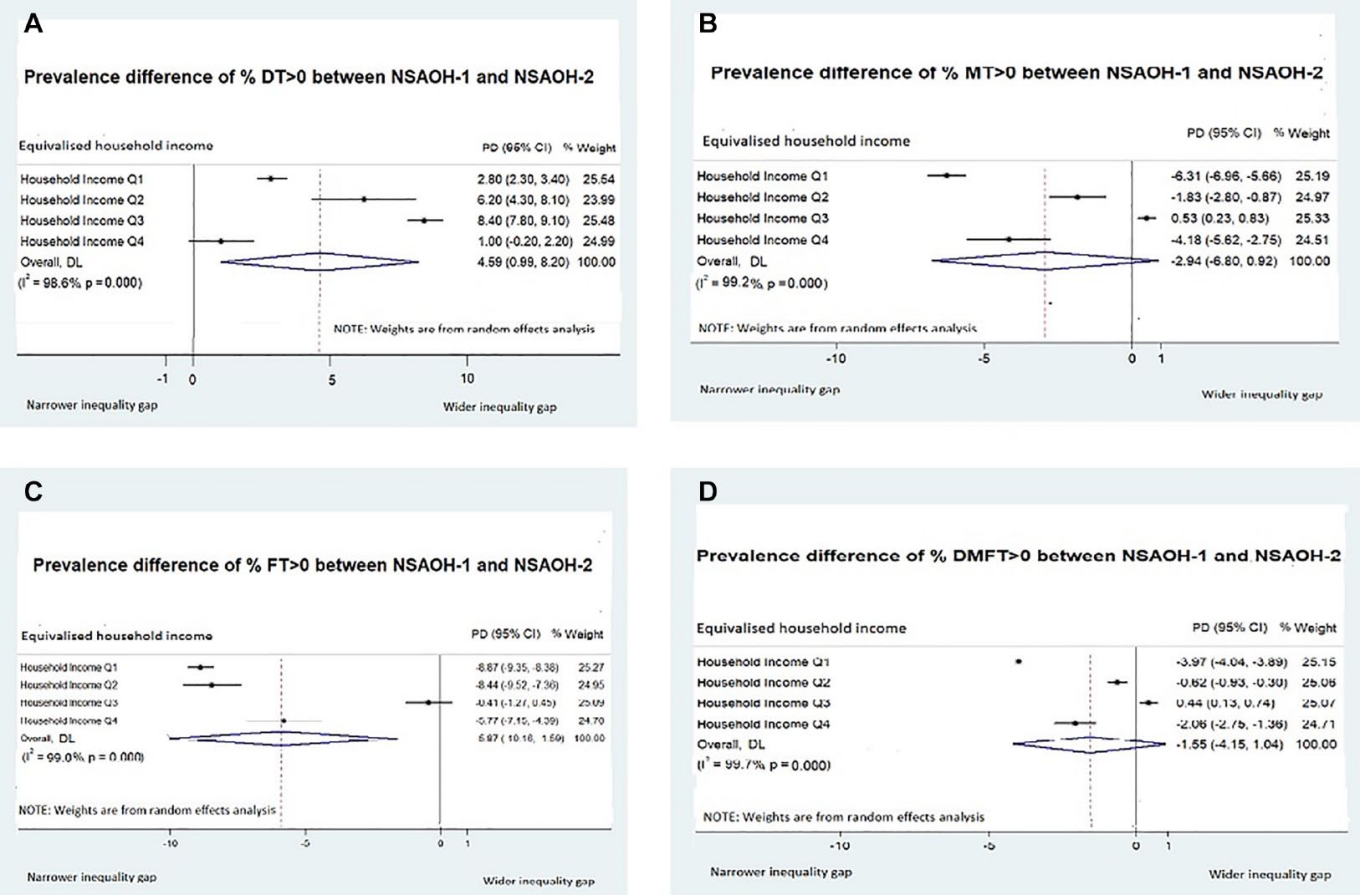
Adjusted prevalence difference (PD) by income status among Australian adults. (**A**) PD for % DT >0. (**B**) PD for % MT >0. (**C**) PD for % FT >0. (**D**) PD for % DMFT >0. DL, DerSimonian Laird random effect; DT, decayed teeth; FT, filled teeth; MT, missing teeth; NSAOH, National Survey of Adult Oral Health.

After adjusting for age and sex, across all income quartiles, % DT >0 was higher in NSAOH-2 than in NSAOH-1 (Table 4). The prevalence increase was most marked in the third highest income group (Q3; PD = 8.4, from 24.1 to 32.5), followed by the second lowest income group (Q2; PD = 6.2, from 26.4 to 32.6), then the lowest income group (Q1; PD = 2.8, from 35.5 to 38.4) then the highest income group (Q4; PD = 1.0, from 19.9 to 20.9). Comparing the lowest to the highest income quartiles, the PD was 15.6 (from 35.5 to 19.9) for NSAOH-1 and 17.5 (from 38.4 to 20.9) for NSAOH-2, indicating that the social inequities between income groups for dental caries increased between the 2 time points (PD = 1.8). Forest plots for the adjusted PD between NSAOH-1 and NSAOH-2 household income groups are shown in Figure 1. For % DT >0, the largest inequity was observed in Q3 (8.4). This was followed by Q2 (6.2), Q1 (2.8), and then the highest income group, Q4 (1.0). The summary estimate for adjusted PD (all income groups combined) was 4.6.

**Table 4.** Adjusted Prevalence and Prevalence Difference (PD) for Clinical Oral Health Characteristics by Income Status.

	% DT >0		% MT >0	
	Prevalence (95% CI)	PD (95% CI)	Prevalence (95% CI)	PD (95% CI)
Household income Q1 (low)				
NSAOH-1	35.5 (30.8 to 40.3)	2.8 (2.3 to 3.4)	74.1 (69.1 to 79.2)	−6.3 (−7.0 to −5.7)
NSAOH-2	38.4 (33.0 to 43.5)	Ref	67.8 (62.2 to 73.5)	Ref
Household income Q2				
NSAOH-1	26.4 (22.9 to 29.9)	6.2 (4.3 to 8.1)	65.8 (61.3 to 70.2)	−1.8 (−2.8 to −0.9)
NSAOH-2	32.6 (27.2 to 38.0)	Ref	63.9 (58.5 to 69.4)	Ref
Household income Q3				
NSAOH-1	**24.1 (20.3 to 28.0)**	8.4 (7.8 to 9.1)	56.5 (51.7 to 61.3)	0.5 (0.2 to 0.8)
NSAOH-2	**32.5 (28.1 to 37.0)**	Ref	57.0 (51.9 to 62.1)	Ref
Household income Q4 (high)				
NSAOH-1	19.9 (16.4 to 23.3)	1.0 (−0.2 to 2.2)	53.2 (48.9 to 57.6)	−4.2 (−5.6 to −2.8)
NSAOH-2	20.9 (16.3 to 25.5)	Ref	49.1 (43.3 to 54.8)	Ref
	% FT >0		% DMFT >0	
	Prevalence (95% CI)	PD (95% CI)	Prevalence (95% CI)	PD (95% CI)
Household income Q1 (low)				
NSAOH-1	81.1 (76.5 to 85.7)	−8.9 (−9.4 to −8.4)	92.9 (89.2 to 96.7)	−4.0 (−4.1 to −3.9)
NSAOH-2	72.2 (67.2 to 77.3)	Ref	89.0 (85.2 to 92.8)	Ref
Household income Q2				
NSAOH-1	85.1 (81.3 to 88.9)	−8.4 (−9.5 to −7.4)	90.5 (87.1 to 93.9)	−0.6 (−0.9 to −0.3)
NSAOH-2	76.6 (71.7 to 81.5)	Ref	89.9 (86.2 to 93.6)	Ref
Household income Q3				
NSAOH-1	82.5 (78.1 to 86.9)	−0.4 (−1.3 to 0.5)	90.0 (87.0 to 92.9)	0.4 (0.1 to 0.7)
NSAOH-2	82.1 (78.6 to 85.6)	Ref	90.4 (87.7 to 93.1)	Ref
Household income Q4 (high)				
NSAOH-1	89.5 (86.7 to 92.3)	−5.8 (−7.2 to −4.4)	92.5 (90.2 to 94.9)	−2.1 (−2.8 to −1.4)
NSAOH-2	83.8 (79.6 to 87.9)	Ref	90.5 (87.4 to 93.5)	Ref

All estimates adjusted for age and sex. 95% CI: confidence interval in parentheses. Estimates in bold denote statistically significant differences. DT, decayed teeth; FT, filled teeth; MT, missing teeth; NSAOH, National Survey of Adult Oral Health; PD, prevalence difference; Ref, reference.

Across all income quartiles, % MT >0 was lower in NSAOH-2 than in NSAOH-1 (with the exception of Q3, with a negligible PD; Table 2). This decrease was most marked for the lowest income group, Q1 (PD = −6.3, from 74.1 to 67.8); followed by the highest income group, Q4 (PD = −4.2, from 53.2 to 49.1); then Q2 (PD = −1.8, from 65.8 to 63.9) and Q3 (PD = 0.5, from 56.5 to 57.0). Comparing the lowest to highest income quartiles, the PD was 20.9 in NSAOH-1 (from 74.1 to 53.2) and 18.7 in NSAOH-2 (from 67.8 to 49.1). This indicates that social inequities in the prevalence of missing teeth decreased between the 2 time periods (PD = −2.2). The summary estimate for adjusted PD for % MT >0 (all income groups combined) was −2.9.

Across all income quartiles, % FT >0 was lower in NSAOH-2 than in NSAOH-1. The decrease was the most marked for the lowest income group, Q1 (PD = −8.9, from 81.1 to 72.2), followed by the Q2 (PD = −8.4, from 85.1 to 76.6), Q4 (PD = −5.8, from 89.5 to 83.8) then Q3 (PD = −0.4, from 82.5 to 82.1). Comparing the lowest to the highest income groups, the PD was 8.4 in NSAOH-1 (from 81.1 to 89.5) and 11.6 in NSAOH-2 (72.2 to 83.8). This indicates the social inequities in the prevalence of filled teeth increased across the 2 time periods (PD = 3.2). The summary estimate for adjusted PD for % FT >0 (all income groups combined) was −5.87.

Across all income quartiles, % DMFT >0 was lower in NSAOH-2 than in NSAOH-1 (with the exception of Q3, which was negligible). The decrease was most marked for the lowest income group, Q1 (PD = −4.0, from 92.9 to 89.0), followed by the highest income group, Q4 (PD = −2.1, from 92.5 to 90.5), then Q2 (PD = −0.6, from 90.5 to 89.9) and Q3 (PD = 0.4, from 90.0 to 90.4). Comparing the lowest to the highest income groups, the PD was −0.4 in NSAOH-1 (from 92.9 to 92.5) and 1.5 in NSAOH-2 (89.0 to 90.5), indicating the social inequities in the prevalence of decayed, missing, and filled teeth increased across the 2 time periods (PD = 1.9). The summary estimate for adjusted PD for % DMFT >0 (all income groups combined) was −1.55.

## Discussion

Our findings describe social differentials in dental caries among Australian adults across a 13-y time period and confirm that although oral health inequities decreased for the most extreme management outcome of dental caries (missing teeth due to dental caries), inequities increased for experience of that disease (dental caries) and the more conservative management of dental caries (filled teeth). For all D, M, and F components (DMFT), inequities between the lowest and highest household income groups increased from 2004–2006 to 2017–2018.

It is difficult to compare our findings with those from other countries because of the limited number of national oral health surveys conducted regularly at a global level that include clinical data. In the United Kingdom, the most recent national oral health surveys that included dental examinations were conducted in 1998 and 2009, with the 2021 data collection containing questionnaire data only (due to COVID-19 restrictions; Office for Health Improvement and Disparities 2023). In the United States, the National Health and Nutrition Examination Survey (NHANES) is a complex, multistage probability sample of the noninstitutionalized US population (Dye et al. 2019) with a range of health outcomes assessed including clinical oral health. NHANES examines a nationally representative sample of about 5,000 persons each year; however, to the best of our knowledge, there has been no reporting of changes in oral health inequities in D, M, and F outcomes. New Zealand conducted a National Oral Health Survey in 2009, but it has not been repeated since (New Zealand Ministry of Health 2010). Canada collects a range of health outcomes at a representative, population level through its Canadian Health Measures Survey; however, clinical oral health data have been collected only once, in 2007–2009 (Lavinge and Allison 2022).

Our findings highlight the importance of differentiating the individual components comprising the DMFT index when assessing changes over time, as when taken together, the effects of, for example, an increased mean DT and prevalence of % DT >0 and a decreased mean MT and prevalence of % MT >0 across the 2 time points cancel each other out. This has been highlighted by others in the field (Meija et al. 2004). These estimates indicate that an annual dental checkup is more important and recognized to reduce dental caries.

About half of Australia’s adult population has access to private dental insurance (ARCPOH 2019), and in the 2017–2019 NSAOH, just more than 80% had last attended a private dentist. Between 2004–2006 and 2017–2018, the percentage who avoided or delayed dental care due to cost increased from 30.6% to 39.2%. Previous studies (Chrisopoulos et al. 2013; Elani et al. 2017; Ju et al. 2021) indicated that inequality in dental care utilization and avoiding dental visiting were associated with inequities between low and high household income groups. Therefore, narrowing or closing the wealth gap is an important link in reducing dental diseases.

Our stratified analyses demonstrate obvious social gradients with respect to experience of dental caries and missing teeth in both 2004–2006 and 2017–2018, with the most socially disadvantaged bearing the greatest burden of disease. More socially advantaged groups had a higher proportion of filled teeth across both time points, indicating both better access to dental care and ability to afford care that supports maintenance of preservation of the dentition and supporting structures. Reasons for tooth extraction likely include extensiveness and severity of disease, treatment philosophy, and cost preventing more restorative treatment options (Dye et al. 2019).

That inequities increased between the most socially advantaged and disadvantaged groups between 2004–2006 and 2017–2018 is concerning. It suggests that models of dental service provision in Australia are increasingly benefitting those who can afford and access the care and who arguably need less services than their less socially advantaged counterparts. Dental care provision through the public sector in Australia is recognized as being underfunded and with complex eligibility requirements (PWC 2019), with all states and territories experiencing profound shortages in workforce (both dentists and oral health therapists; Australian Institute of Health and Welfare 2023). Without a fundamental change in the funding models and workforce, these inequities are likely to increase. Promoting the level of education and improving the level of dental health literacy at a population level to increase the awareness of prevention of dental diseases is also crucial (Ju et al. 2021).

## Author Contributions

L.M. Jamieson, G.C. Mejia, contributed to conception and design, data acquisition and interpretation, drafted and critically revised the manuscript; L. Luzzi, contributed to conception, data acquisition, drafted and critically revised the manuscript; S. Chrisopoulos, contributed to design, data interpretation, drafted and critically revised the manuscript; X. Ju, contributed to conception and design, data acquisition, analysis, and interpretation, drafted and critically revised the manuscript. All authors gave their final approval and agreed to be accountable for all aspects of the work.
